# HDAC3 inhibition alleviates neuroinflammation and white matter injury after subarachnoid hemorrhage through modulation of BDNF/TrkB/NF-κB signaling

**DOI:** 10.1016/j.neurot.2026.e01099

**Published:** 2026-10-28

**Authors:** Hongqiao Yang, Ziliang Hu, Hao Zhuang, Siqi Chen, Shengjun Zhou, Meng Sun, Lifei Lian, Chenhui Zhou, Xiang Gao, Yi Huang

**Affiliations:** a Ningbo Key Laboratory of Nervous System and Brain Function, Department of Neurosurgery, The First Affiliated Hospital of Ningbo University, Ningbo, Zhejiang, 315010, China; b Department of Neurology, Tongji Hospital, Tongji Medical College, Huazhong University of Science and Technology, Wuhan, China

**Keywords:** HDAC3, Neuroinflammation, White matter injury, Subarachnoid hemorrhage, Single-cell RNA sequencing

## Abstract

White matter injury (WMI) is a major contributor to neurological deficits after subarachnoid hemorrhage (SAH). Histone deacetylase 3 (HDAC3) is an established modulator of inflammatory responses, yet its contribution to post-SAH white matter pathology remains poorly understood. We used a rat endovascular perforation model and treated animals with RGFP966, a selective HDAC3 inhibitor. Clinical data, single-cell RNA sequencing (scRNA-seq), magnetic resonance imaging (MRI), and transmission electron microscopy were integrated to evaluate cellular remodeling and structural integrity. Short- and long-term neurological recovery was assessed using a systematic battery of behavioral tests, while the underlying BDNF/TrkB/NF-κB signaling axis was validated through gain- and loss-of-function experiments. HDAC3 levels were elevated in both the CSF of SAH patients and rat brain tissues, inversely correlating with myelin basic protein (MBP) expression. RGFP966 treatment effectively preserved WMI, evidenced by reduced axonal injury and improved myelin shielding, and translated to superior long-term cognitive performance. ScRNA-seq analysis identified a profound shift in the microglial and oligodendrocyte-lineage landscapes after SAH. HDAC3 inhibition effectively curbed the expansion of pro-inflammatory microglial subsets while restoring homeostatic signatures and enhancing pro-myelination programs. Mechanistically, RGFP966 upregulated BDNF expression and restored TrkB phosphorylation, thereby suppressing NF-κB-mediated neuroinflammation. Notably, BDNF knockdown abolished the protective effects of HDAC3 inhibition, whereas exogenous BDNF mimicked the anti-inflammatory and myelin-protective phenotype induced by RGFP966. In conclusion, our findings demonstrate that HDAC3 is a pivotal driver of post-SAH neuroinflammation and WMI. Inhibition of HDAC3 exerts neuroprotection by recalibrating the microglial immune microenvironment and restoring the BDNF/TrkB axis.

## Introduction

Subarachnoid hemorrhage (SAH) accounts for 5%–7% of all strokes, leading to high mortality rates and significant long-term disability, with up to 20% of survivors left permanently impaired [1], [2]. Traditional studies have focused on early protection of cortical or hippocampal neuronal cell bodies, whereas white matter injury (WMI) is often overlooked. Clinical studies have shown that patients with SAH exhibit widespread white matter abnormalities, which are closely associated with long-term cognitive and motor deficits [3]. Consistently, experimental SAH models demonstrate early white matter damage characterized by myelin loss, axonal disruption, and white matter edema [4], [5]. Because white matter tracts are essential for interregional communication, cognitive processing, and sensorimotor integration, injury to the axon - myelin unit may represent an important structural basis for the sustained functional deficits observed after SAH. Therefore, developing a series of therapeutic strategies targeting WMI could help improve the long-term prognosis following SAH.

Neuroinflammation is increasingly recognized as a central mechanism underlying WMI after SAH. Among the cellular mediators involved, microglia are rapidly activated after hemorrhagic insult and play a pivotal role in shaping the post-SAH inflammatory microenvironment [5], [6], [7]. Excessive or sustained microglial activation promotes the release of pro-inflammatory cytokines, reactive oxygen species, and other injury-associated mediators, thereby aggravating secondary brain injury (SBI) and contributing to myelin and axonal damage [8]. Although microglial responses have traditionally been described within the framework of M1 and M2 polarization, recent evidence indicates that microglial activation states in central nervous system (CNS) injury are substantially more heterogeneous and dynamic than this binary classification suggests [9]. Nevertheless, inflammation-dominant microglial responses are generally considered detrimental, whereas more homeostatic or reparative phenotypes may help limit tissue injury and promote repair [6], [7]. Accordingly, modulation of maladaptive microglial activation has emerged as a potential strategy for attenuating neuroinflammation and white matter damage after SAH [10], [11].

Histone deacetylase 3 (HDAC3) is a critical epigenetic regulator involved in neurodevelopment, repair, and pathological processes [12]. Recent studies have identified HDAC3 as an important mediator of neuroinflammation and white matter pathology in multiple neurological disorders. In traumatic brain injury, microglia-specific deletion of HDAC3 suppresses neuroinflammation, reduces axonal and myelin injury, and improves long-term functional recovery [13]. In ischemic stroke, HDAC3 inhibition has also been reported to facilitate white matter repair and functional improvement [14]. These findings suggest that HDAC3 may represent a convergent regulator linking inflammatory activation to white matter damage. However, whether HDAC3 contributes to neuroinflammation and WMI after SAH, and how it influences injury-related cellular responses in this context, remain unclear.

Brain-derived neurotrophic factor (BDNF) and its receptor TrkB are key regulators of neuronal survival, synaptic plasticity, and glial homeostasis, and have also been implicated in myelin maintenance and repair after CNS injury [15]. In parallel, NF-κB signaling is a central driver of inflammatory gene transcription and has been strongly linked to post-SAH neuroinflammation [16]. Previous studies suggest that HDAC3 can modulate neuroprotective and inflammatory gene programs, but whether HDAC3 regulates SAH-induced neuroinflammation and WMI through the BDNF/TrkB/NF-κB axis has not been determined. Moreover, conventional bulk-level approaches are insufficient to resolve the cellular heterogeneity and dynamic state transitions that occur after SAH. Single-cell RNA sequencing provides an opportunity to dissect these complex cellular responses at high resolution and to define how HDAC3 inhibition reshapes injury-associated transcriptional programs across distinct cell populations.

In the present study, we combined pharmacological inhibition of HDAC3 with single-cell transcriptomic profiling to investigate the role of HDAC3 in neuroinflammation and WMI after SAH. We found that HDAC3 inhibition alleviated white matter damage, suppressed maladaptive microglial inflammatory activation, and reshaped oligodendrocyte-lineage responses after SAH. Mechanistically, these protective effects were associated with restoration of BDNF/TrkB signaling and suppression of NF-κB activation. Our findings identify HDAC3 as a previously underappreciated regulator of SAH-induced neuroinflammation and WMI, and suggest that targeting HDAC3 may represent a promising therapeutic strategy for improving long-term neurological outcomes after SAH.

## Materials and Methods

### Study population and sample collection

Cerebrospinal fluid (CSF) samples were collected from 50 neurosurgical inpatients at the First Affiliated Hospital of Ningbo University. Of these, 40 patients with SAH were included in the disease group, and 10 patients with trigeminal neuralgia were served as controls. The inclusion criteria were aged ≥18 years and a diagnosis of SAH confirmed by head CT. The exclusion criteria were: (1) incomplete or missing critical clinical data (e.g., absence of an initial CT examination within 72 h after SAH); (2) infections occurring before or after SAH onset, such as pulmonary or intracranial infections; and (3) severe mental or cognitive impairments that hindered cooperation with the examination. Diagnoses were independently made by at least two experienced neurosurgeons. CSF samples were collected within 7 days of admission after written informed consent had been obtained. This study was approved by the Ethics Committee of the First Affiliated Hospital of Ningbo University (Approval No: 2024113 A), and written informed consent was obtained from all participants.

### Animals

All experimental procedures in this study were in accordance with ethical guidelines for animal research and were approved by the Ethics Committee of the Ningbo University Laboratory Animal Center. Male SD rats weighing 280–300 g were obtained from the Ningbo University Animal Center. The rats were housed under controlled conditions at room temperature (22 ± 1 °C) and constant humidity (60 ± 5%), with a 12-h light/dark cycle. Food and water were provided ad libitum.

### SAH animal model

The endovascular perforation method was used to develop the rat model of SAH following established protocols [11]. Specifically, the animals were subjected to general anesthesia, induced by 4 % isoflurane in 100 % oxygen within a transparent acrylic chamber, followed by maintenance with 2 % isoflurane and analgesia (buprenorphine: 0.1 mg/kg, s. c.). A heating pad was utilized to keep the animals’ body temperature at 37 ± 0.5 °C. A midline incision was made on the neck, and subcutaneous tissue was carefully separated in layers to expose the right external carotid artery, which was then isolated and ligated using an electrocautery device. A 6-0 silk suture was employed to temporarily occlude blood flow. A small incision was made in the external carotid artery using microsurgical scissors, through which a single nylon suture was advanced into the cranial cavity, directed toward the bifurcation of the anterior cerebral artery and middle cerebral artery. The suture was then further advanced by 2 mm to puncture the vessel. Following this, blood flow was restored to induce SAH. Rats in the sham surgery group underwent the same surgical procedure, excluding the vessel puncture.

### Drug administration

RGFP966, a selective HDAC3 inhibitor, was prepared in a vehicle containing 10% DMSO and 90% corn oil. Based on previous studies [17], [18], RGFP966 was administered intraperitoneally at a 10 mg/kg after SAH induction, the Sham and SAH groups received an equivalent volume of vehicle. For long-term assessment, RGFP966 (10 mg/kg) was administered intraperitoneally for 5 consecutive days starting 1 day after SAH [19]. Recombinant human BDNF (rhBDNF) was prepared in phosphate-buffered saline (PBS) with a concentration of 0.5 μg/μL and was administered intraventricularly 30 min after SAH induction. PBS was used as the vehicle control. Lentiviral BDNF shRNA and the corresponding empty vector were purchased from GenePharma (Shanghai, China), with an injection rate of 0.5 μL/min. A total of 2 μL of lentivirus was injected intraventricularly 72 h prior to SAH induction.

### Experimental design

The experimental design and animal groups are shown in Fig. 1, rats were randomly assigned to six separate experimental groups. Rats with the SAH Grade score greater than or equal to 9 points were included in the study, and the score less than 9 points and dead rats were excluded from the experiment. Repeat the procedure until sufficient sample size is achieved for statistical analysis.

**Fig. 1:**
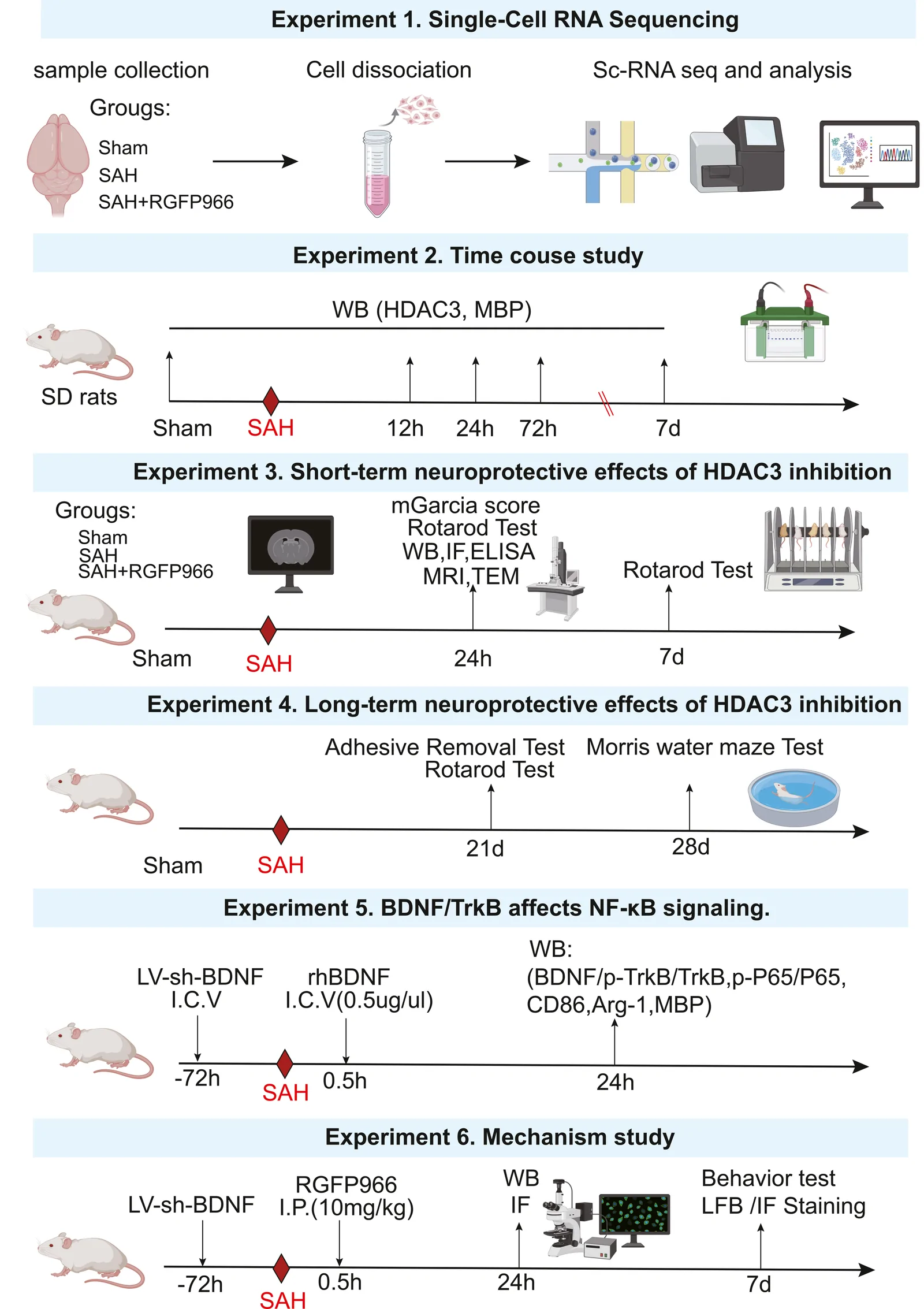
Schematic representation of experimental design and subsequent analyses.

#### Experiment 1. single-cell RNA sequencing

The experimental animals were randomly divided into 3 groups, namely the Sham group, the SAH group, and the SAH + RGFP966 group. One animal per group was used for single-cell RNA sequencing. Samples were collected 24 h after SAH from the injured cortex and peri-corpus callosum region.

#### Experimental 2. time course study

To characterize the temporal changes of HDAC3 and MBP expression in the rat brain after SAH, animals were randomly assigned to five groups: Sham (n = 6), SAH12h (n = 6), SAH24h (n = 6), SAH72h (n = 6), and SAH7d (n = 6). Western blotting (WB) was performed to determine HDAC3 and MBP protein levels at the indicated time points. Tissue samples were obtained from the injured cortex and peri-corpus callosum region.

#### Experimental 3. short-term neuroprotective effects of HDAC3 inhibition

To explore Short-term neuroprotective effects of HDAC3 inhibition on acute neuroinflammation and white matter damage, animals were randomly assigned to three groups: Sham (n = 12), SAH (n = 12), and SAH + RGFP966 (n = 12). RGFP966, a selective HDAC3 inhibitor, was administered 30 min after SAH. Neurological function was assessed using the modified Garcia (mGarcia) score at 24 h after surgery, and motor coordination and balance were evaluated using the rotarod test at 24 h and 7 d after surgery. Magnetic resonance imaging (MRI) was used to quantify the relative extent of brain tissue injury. Western blot (WB) (n = 6) was used to assess protein expression, Immunofluorescence (IF) staining (n = 3) was used to evaluate Iba-1, MBP and microglial phenotypes, transmission electron microscopy (TEM) (n = 3) was used to assess myelin and axon loss, and Enzyme-Linked Immunosorbent Assay (ELISA, n = 3) was used to measure inflammatory mediators. The assessments were perfomed 24 h after injury, and WB and ELISA analyses used shared tissue samples.

#### Experiment 4. long-term neuroprotective effects of HDAC3 inhibition

To investigate the effects of HDAC3 inhibition on long-term neurological and cognitive outcomes after SAH, animals were randomly assigned to three groups: Sham (n = 16), the SAH (n = 16), and the SAH + RGFP966 (n = 16). Sensorimotor function was evaluated using the rotarod and adhesive removal tests on day 21 after surgery. Long-term spatial learning and memory were assessed using the Morris water maze from days 24–28 after surgery.

#### Experiment 5. BDNF/TrkB affects NF-κB signaling

This experiment was designed to determine whether BDNF/TrkB signaling regulates NF-κB activation, microglia-associated neuroinflammation, and MBP expression. Animals were randomly assigned to four groups: Sham (n = 6), SAH + Veh (n = 6), SAH + rhBDNF (n = 6), and SAH + shBDNF (n = 6). LV-shBDNF was administered 72 h before SAH, whereas rhBDNF was administered 30 min after injury. Protein expression was assessed by WB.

#### Experiment 6. mechanism study (BDNF/TrkB/NF-κB signaling pathway)

To investigate whether HDAC3 inhibition exerts neuroprotective effects through the BDNF/TrkB/NF-κB signaling pathway, rats were randomly assigned to four groups: SAH (n = 15), SAH + RGFP966 (n = 15), SAH + RGFP966+sh-NC (n = 15), and SAH + RGFP966+sh-BDNF (n = 15). WB and IF analyses were performed at 24 h after surgery. Behavioral tests, LFB staining and IF were performed 7 days after SAH.

### Single-cell RNA sequencing and analysis

#### Single-cell RNA sequencing

To characterize cellular heterogeneity and transcriptional alterations in the brains of rats from the Sham, SAH, and SAH + RGFP966 groups, single-cell RNA sequencing (scRNA-seq) was performed. At 24 h after surgery, cortical tissue together with the peri-corpus callosum region was harvested and dissociated into single-cell suspensions according to standard procedures [5]. Briefly, brain tissues were mechanically minced and enzymatically digested at 37 °C for 30 min in a digestion buffer containing normal DMEM, 15 mg/mL collagenase type II, and 500 μg/mL elastase. Cell debris and myelin were subsequently removed, and viable cells were enriched before library preparation. Cell viability was assessed before sequencing, and only samples meeting the viability criteria were included in downstream analyses. Single-cell libraries were generated using the 10x Genomics Chromium Single Cell 3′ platform and sequenced on an Illumina NovaSeq 6000 platform according to the manufacturer's instructions [20].

#### Cell Ranger processing

Sequencing-derived FASTQ files were processed using Cell Ranger single-cell software (version [10.0.0]) for demultiplexing, alignment, filtering, barcode assignment, unique molecular identifier (UMI) counting, and gene expression quantification for each sample, following the 10x Genomics guidelines. Reads were aligned to the rat reference genome (*Rattus norvegicus*) [20].

#### Quality control, normalization, and scaling

Downstream analyses were performed in R using the Seurat package (version 5.4.0). Raw UMI count matrices were imported and converted into Seurat objects. Cells were retained as high-quality cells if they met the following criteria: detected gene number >200, UMI count >1,000, log10GenesPerUMI >0.7, mitochondrial UMI proportion <15%, and hemoglobin gene proportion <5%. Putative doublets were identified and removed using the DoubletFinder package. Cell-cycle phase scores were calculated using the CellCycleScoring function in Seurat based on canonical S-phase and G2/M-phase gene sets. Cell-cycle distribution was evaluated as part of quality assessment, but cell-cycle effects were not regressed out in downstream analyses.

After quality control, the data were normalized and scaled. Global-scaling normalization was performed using the “LogNormalize” method with a scale factor of 10,000. The top 2000 highly variable genes in each dataset were identified using the variance-stabilizing transformation (vst) method [21].

#### Dimensionality reduction, clustering, and batch correction

To reduce potential batch effects across samples, Harmony was used for data integration. Principal component analysis (PCA) was performed for dimensionality reduction of the integrated dataset, and the top 10 principal components were used for downstream analyses. Unsupervised clustering was performed using the “FindNeighbors” and “FindClusters” functions. The resolution parameter was evaluated across a range of 0.1–2.0, and a resolution of 1.0 was ultimately selected for downstream analyses and visualization. Uniform manifold approximation and projection (UMAP) was used for two-dimensional visualization.

#### Cell annotation

Cell clusters were annotated based on canonical marker genes and established cell-type signatures. Marker genes were obtained from previous studies and the CellMarker database (http://117.50.127.228/CellMarker/) [9], [22], [23]. Major cell populations were first identified at the whole-tissue level. Microglia and oligodendrocyte-lineage cells were then extracted and re-clustered separately for further analyses. Microglial subpopulations were annotated as homeostatic, inflammatory, stressed, phagocytic, and ribosome biogenesis states [9]. Oligodendrocyte-lineage cells were classified into oligodendrocyte precursor cells (OPCs), newly formed oligodendrocytes, and mature oligodendrocytes according to canonical markers.

#### Enrichment analysis and functional state scoring

Differentially expressed genes (DEGs) between groups or subclusters were identified using the default Wilcoxon rank-sum test implemented in Seurat. Genes with |log 2 fold change (FC)| > 0.58 (FC > 1.5) and adjusted P < 0.05 were considered significantly differentially expressed. Functional enrichment analyses of DEGs were performed using the clusterProfiler package based on the Kyoto Encyclopedia of Genes and Genomes (KEGG) and Gene Ontology (GO) databases. In addition, gene set enrichment analysis (GSEA) was performed to assess pathway-level changes associated with inflammatory signaling and myelination-related programs. The normalized enrichment score (NES) was calculated for each pathway, and gene sets with |NES| > 0.5, false discovery rate (FDR) q value < 0.05, and nominal P value < 0.05 were considered significantly enriched.

To quantify functional states at the single-cell level, inflammatory response scores and myelination-related scores were calculated using AddModuleScore or AUCell based on predefined gene sets. These analyses were used to compare inflammatory activation across microglial subpopulations and to evaluate myelination-associated transcriptional programs in oligodendrocyte-lineage cells. Heatmaps of pathway activity, violin plots, dot plots, stacked bar plots, and UMAP visualizations were generated in R using Seurat and related packages.

#### Pseudotime and cellchat analyses

Pseudotime trajectory analysis was performed using Monocle 2 to investigate dynamic cell-state transitions [24], [25]. Highly variable genes were selected as ordering genes, and dimensionality reduction and trajectory construction were performed using the DDRTree algorithm implemented in the reduceDimension function. Pseudotime trajectories were visualized using plot_cell_trajectory, with cells colored according to cell type and state.

Cell–cell communication analysis was performed using CellChat (v2.1.0) [26]. Communication probabilities were inferred using the computerphobe function with the triMean method, and cell populations containing fewer than 10 cells were excluded. Pathway-level communication probabilities were calculated using computeCommunProbPathway and aggregated with aggregateNet. Intercellular signaling networks were visualized using CellChat visualization functions, including netVisual_bubble.

### SAH grade

The SAH grading score was assessed 24 h after SAH by an investigator who was blinded to the experimental group assignments, as described previously [27]. In brief, the basal cisterns were divided into six segments, with each segment assigned a grade from 0 to 3. Grades 0, 1, 2, and 3 correspond to the absence of a noticeable subarachnoid blood clot, a small blood clot, a moderate blood clot, and a large blood clot that obscures the Circle of Willis, respectively. Rats with a grade of <8 at 24 h post-SAH were excluded from the study.

### Neurological function assessment

#### mGarcia score

As described previously [28], neurological function was evaluated using the mGarcia scoring system, which consists of six components: spontaneous activity (0–3), symmetry of limb movements (0–3), body proprioception (1–3), forelimb extension (0–3), climbing ability (1–3), and response to vibrissae touch (1–3).

#### Rotarod test

As described previously [29], the rotarod test was performed to evaluate motor coordination and balance in rats on days 1, 7, and 21 after SAH. The rotation speed was initially set at 4 rpm and gradually increased at a rate of 0.5 rpm/s. The latency to fall from the accelerating rotating rod was recorded.

#### Adhesive removal test

To assess sensorimotor function of the forelimbs, the adhesive removal test was performed as previously described [30]. Briefly, adhesive tapes were applied to the forelimbs of each rat. The time taken to respond to the presence of the adhesive tape and the time required to successfully remove the tape were recorded.

#### Morris water maze test

The Morris water maze test was used to evaluate spatial learning and memory as previously described [30], [31]. The pool (160 cm in diameter) was filled with water and made opaque. A hidden platform was submerged 1 cm below the water's surface in one quadrant, with its position remaining constant throughout the test. Rats underwent 4 trials per day for 5 consecutive days, with each trial allowing up to 60 s for the animal to locate the platform. If the rat did not find the platform within 60 s, it was guided to the platform and allowed to remain there for 30 s. The time taken to find the platform (escape latency) was recorded. On the final day, a probe trial was conducted, where the platform was removed, and the rat was allowed to swim for 60 s. The swim path, number of platform entries, escape latency, and time spent in the target quadrant were recorded using a computerized tracking system (Noldus Ethovision; Noldus, Tacoma, WA, USA).

### ELISA

Human CSF levels of HDAC3, MBP, and rat brain of inflammatory factors (TNF-α, IL-6, IL-1β) were quantified by ELISA according to the manufacturers' instructions. The ELISA kits for TNF-α, IL-6, IL-1β, and MBP were purchased from Elabscience (Rat TNF-α ELISA Kit: E-EL-R2856; Rat IL-6 ELISA Kit: E-EL-R0015; Rat IL-1β ELISA Kit: E-EL-R0012; Human MBP ELISA Kit, Cat. No. E-EL-H0161; all from Elabscience, Wuhan, China). The HDAC3 concentration was measured using a validated ELISA assay following the kit's protocol (LS-F55924, LS Bio, USA).

### Luxol fast blue (LFB) staining

LFB staining was performed according to a previously described protocol [32]. Briefly, prepared brain sections were rinsed with distilled water and treated with 95% ethanol, followed by incubation with LFB staining solution. After staining, excess dye was removed with ethanol, and the sections were rinsed with distilled water. The sections were then differentiated using LFB differentiation solution, followed by further differentiation in ethanol until a clear distinction between the gray and white matter was achieved. Finally, the sections were washed, dehydrated, cleared, and mounted. Images were acquired using a light microscope.

### MRI

After anesthesia, the rats was fixed in place with its head and limbs secured. T2-weighted anatomical images of the skull were acquired using an MRI scanner (Bruker, Germany), with high-signal areas representing the injury regions. The images were analyzed using Anaconda Navigator software to calculate the lesion area for each slice and the corresponding brain tissue area. The average lesion area was divided by the average brain tissue area to obtain the ratio of total lesion area to total brain area for each rat.

### TEM

Rats were anesthetized and perfused with PBS followed by an electron microscopy fixative (2% PFA, 2% glutaraldehyde). The ipsilateral corpus callosum was dissected into 1 mm sections and fixed in 2.5% glutaraldehyde for 24 h, followed by fixation in 1% osmium tetroxide for 1 h. The tissue underwent gradient dehydration and resin embedding, after which 70 nm thick sections were cut using a Leica UC7 ultramicrotome, placed on grids, and stained with uranyl acetate and lead citrate. Myelin images were obtained with a transmission electron microscope (Hitachi, HT7800, Japan) and analyzed using Image J software.

### IF staining

After brain tissue was extracted, it was fixed in a tissue fixative solution for 24 h. The tissue was then subjected to a sucrose gradient for dehydration. Following dehydration, the tissue was embedded in OCT and used for frozen sectioning. Coronal sections were cut at the midline of the corpus callosum, with a thickness of 10 μm. The sections were permeabilized with 0.3% Triton X-100 (Beyotime, ST797), followed by washing with PBS. To block nonspecific binding, 5% BSA (Beyotime, C0265) was applied. After blocking, primary antibodies were incubated overnight at 4 °C. The sections were then washed with PBS and incubated with secondary antibodies in the dark at room temperature for 1 h. Following secondary antibody incubation, the slides were washed with PBS, excess moisture was removed, and DAPI staining solution (Beyotime, P0131) was applied for 5 min at room temperature in the dark. After washing with PBS, slides were mounted with an anti-fade mounting medium. The slides were air-dried in the dark and images were captured using a confocal laser scanning microscope (Zeiss, LSM780) and analyzed by ImageJ software. The antibodies used were: mouse anti-IBA-1 (1:200, Millipore, OP80), mouse anti-MBP (1:200, Abcam, ab104224), rabbit anti-IL-1β (1:200, Abcam, ab177487), rabbit anti-Arg-1 (1:200, Abcam, ab75973), Goat anti-rabbit Cy3 (1:200, Proteintech, SA00009-2), and Goat anti-mouse Alexa Fluor 488 (1:200, Abcam, ab150113).

### WB

WB analysis was performed as previously described [33]. Proteins were extracted from ipsilateral cortex and corpus callosum, equal amounts of a sample protein (30 μg) were loaded onto an SDS-PAGE gel. After electrophoresis, the samples were transferred onto a PVDF membrane. Then, the membrane was blocked for 2 h at room temperature and incubated overnight at 4 °C with the following primary antibodies: anti-HDAC3 (1:500, Abcam, Cambridge, MA, USA), anti-Iba-1 (1:1000, Wako, USA), anti-myelin basic protein (MBP, 1:1000, Abcam, Cambridge, MA, USA), anti-BDNF (1:1000, Abcam, Cambridge, MA, USA), anti-phospho-TrkB (1:2000, Abcam, Cambridge, MA, USA), anti-TrkB (1:1000, Cell signaling, USA), anti-phospho-P65 (1:1000, Cell signaling, Danvers, MA, USA), anti-P65 (1:1500, Santa Cruz, Dallas, TX, USA), anti-Arg-1 (1:500, Abcam, Cambridge, MA, USA), anti-CD86 (1:1500, Santa Cruz, Dallas, TX, USA), and anti-β-actin (1:5000, Santa Cruz, Dallas, TX, USA). Appropriate secondary antibodies (1:5000, Santa Cruz, Dallas, TX, USA) were selected to incubate with the membrane for 2 h at room temperature. Then, blot bands were visualized with an ECL reagent (Amersham Biosciences UK Ltd., PA, USA). Non-saturated bands were selected to perform densitometry quantification using Image J software (Image J 1.51, NIH, USA).

### Statistical analysis

Data are presented as the mean ± standard deviation (SD), and statistical analysis was conducted using GraphPad Prism 9.3.1 (GraphPad Software, USA). Normality was assessed using the Shapiro-Wilk test, and homogeneity of variances was evaluated with the Brown-Forsythe test. Differences between two groups were determined by Student's t-test (two-tailed). For comparisons among multiple groups, one-way or two-way ANOVA was used. A p-value of <0.05 was considered statistically significant.

## Results

### Animal usage and mortality

As shown in Table 1, a total of 239 male rats were used in this present study. Among them, 204 rats underwent SAH surgery, whereas 35 rats underwent sham surgery. The overall mortality rates were 14.7% (30/204) and 0% (0/35) in the SAH and sham groups, respectively. A total of 14 SAH rats were excluded due to the mild SAH grade (SAH grade <9) or death during surgery (n = 30).

**Table 1:** Animal usage and mortality.

Groups	Mortality (n)	Excluded
**Part 1:Single cell RNA sequencing**		
Sham (n = 1);	0.0% (0/1)	**0**
SAH (n = 1);	0.0% (0/1)	**0**
SAH + RGFP966 (n = 1)	0.0% (0/1)	**0**
**Part 2: Time course study**		
Sham (n = 6)	0.0% (0/6)	0
SAH (12 h, 24 h, 72 h, 7 d) (n = 24)	13.7% (4/29)	1
**Part 3: Short-term neuroprotective effects of HDAC3 inhibition**		
Sham (n = 12)^a^	0.0% (0/12)	0
SAH + Vehicle1 (n = 12)	20.0% (3/15)	0
SAH + RGFP966 (10 mg/kg) (n = 12)	14.3% (2/15)	1
**Part 4: Long-term neuroprotective effects of HDAC3 inhibition**		
Sham (n = 16)	0.0% (0/16)	0
SAH + Vehicle1 (n = 16)	20.0% (4/20)	0
SAH + RGFP966 (n = 16)	15.8% (3/19)	0
**Part 5: BDNF/TrkB affects NF-**κ**B signaling**		
Sham (n = 6)^a^	0.0% (0/6)	0
SAH + Vehicle 2 (n = 6)	14.3% (1/7)	0
SAH + rhBDNF (n = 6)	12.5% (1/8)	1
SAH + sh-BDNF (n = 6)	12.5% (1/8)	1
**Part 6:Mechanism study (BDNF/TrkB/NF-κB signaling pathway)**		
SAH **+** Vehicle1 (N=15)	15.0% (3/20)	2
SAH + RGFP966 (N=15)	10.5% (2/19)	2
SAH + RGFP966+sh-NC (N=15)	14.3% (3/21)	3
SAH + RGFP966+sh-BDNF (N=15)	14.3% (3/21)	3
**Total**		
Sham	0.0% (0/35)	0
SAH	14.7% (30/204)	14

a Means shared.

### Single-cell transcriptomic profiling reveals HDAC3 inhibition-induced remodeling of the cellular landscape after SAH

Single-cell RNA sequencing was performed to characterize the cellular landscape of the brain in the Sham, SAH, and SAH + RGFP966 groups. UMAP analysis identified 11 major cell populations, including microglia, endothelial cells (ECs), oligodendrocytes, neurons, smooth muscle cells (SMCs), monocytes/macrophages (Mono/Mac), neutrophils, astrocytes, T/NK cells, oligodendrocyte precursor cells (OPCs), and fibroblasts (Fig. 2A). Cell proportion analysis revealed marked alterations in cellular composition after SAH (Fig. 2B). Compared with the Sham group, SAH increased the proportions of immune-related populations, particularly Mono/Mac and neutrophils, while reducing the proportion of oligodendrocytes. Cell-type annotation was supported by the expression of canonical marker genes (Fig. 2C). Notably, RGFP966 treatment partially reversed these SAH-induced alterations in cellular composition (Fig. 2B–D). Collectively, these findings demonstrate that SAH induces broad alterations in resident and infiltrating cell populations, whereas HDAC3 inhibition partially counteracts this injury-associated cellular remodeling.

**Fig. 2:**
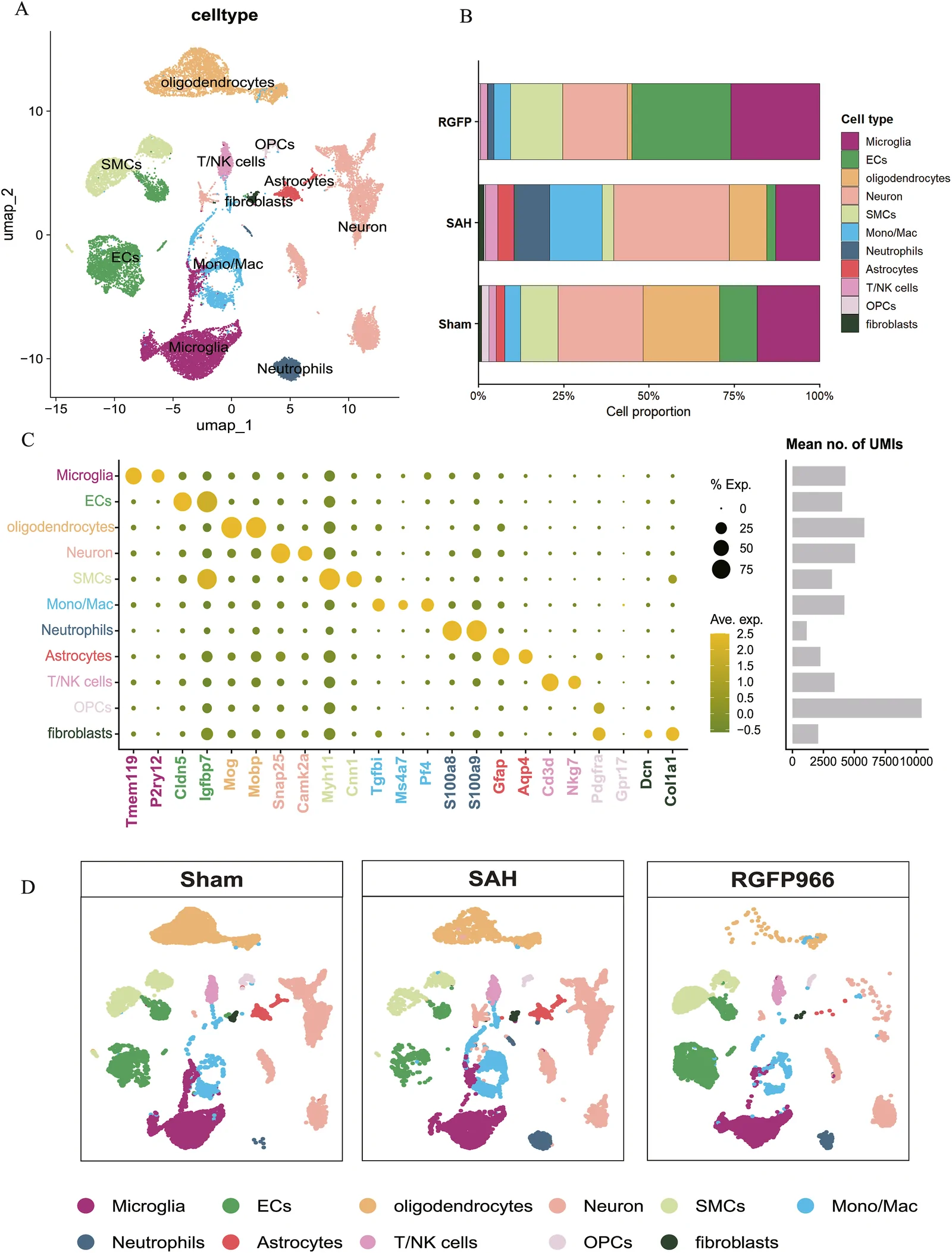
**Single-cell transcriptomic profiling reveals HDAC3 inhibition-induced remodeling of the cellular landscape after SAH** (A) UMAP plot showing the major cell populations identified by single-cell RNA sequencing, including microglia,endothelial cells (ECs), oligodendrocytes, neurons, smooth muscle cells (SMCs), monocytes/macrophages (Mono/Mac), neutrophils, astrocytes, T/NK cells, oligodendrocyte precursor cells (OPCs), and fibroblasts. (B) Stacked bar plot showing the relative proportions of major cell populations in the different three group (C) Dot plot showing the expression of representative marker genes used for cell type annotation across major clusters. Dot size indicates the percentage of cells expressing the gene, and color indicates the average expression level. Bar plot on the right shows the mean number of UMIs in each cell population. (D) Group-wise UMAP plots showing the distribution of major cell populations in the different three groups.

### HDAC3 is associated with SAH and its inhibition improves short- and long-term neurological outcomes in rats

HDAC3 levels in human cerebrospinal fluid increased during days 1–3 of SAH and decreased during days 4–7, but remained higher than those in the control group (Fig. 3 A). In contrast, MBP levels decreased during days 1–3 and increased during days 4–7, but remained lower than those in the control group (Fig. 3 B). HDAC3 and MBP in rat brain tissue showed similar temporal patterns (Fig. 3C–D). SAH grading showed that the severity of hemorrhage was comparable among the SAH groups (Fig. 3E–F). The mGarcia score and rotarod test showed that RGFP966 ameliorated short-term neurological deficits after SAH (Fig. 3G–H). Morris water maze testing further showed that HDAC3 inhibition significantly improved cognitive performance at 28 days after SAH (Fig. 3 I). Specifically, compared with the SAH group, RGFP966 treatment reduced escape latency (Fig. 3J) and increased both the number of platform crossings and the time spent in the target quadrant (Fig. 3K–L).

**Fig. 3:**
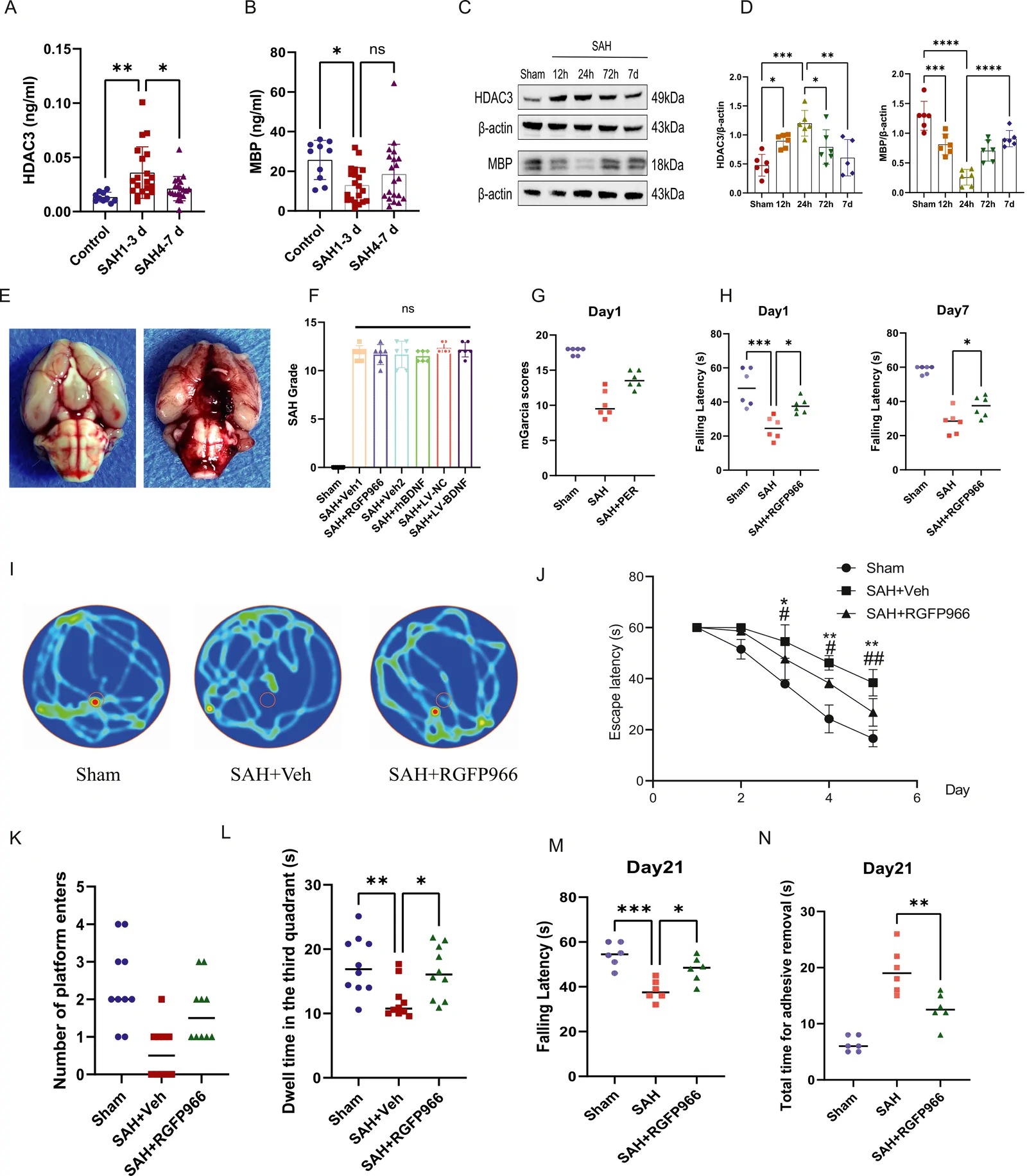
**HDAC3 is associated with SAH and its inhibition improves short- and long-term neurological outcomes in rats** (A, B) Levels of HDAC3 (A) and MBP (B) in cerebrospinal fluid (CSF) samples from control subjects and patients with SAH at 3 and 7 days after onset. (C, D) Representative Western blot images and quantitative analysis of HDAC3 and MBP expression at different time points after SAH in rats. (E) Representative images of brains from Sham and SAH rats. (F) SAH severity scores among the experimental groups. (G) Modified Garcia (mGarcia) scores at day 1 after SAH. (H) Rotarod test showing the latency to fall at days 1 and 7 after SAH. (I) Representative heatmaps of the Morris water maze probe test in the Sham, SAH + Veh, and SAH + RGFP966 groups. (J) Escape latency during the Morris water maze training period (∗P < 0.05 and ∗∗P < 0.01 vs. Sham; #P < 0.05 and ##P < 0.01 vs SAH + Veh.). (K) Number of platform-area entries during the probe test. (L) Time spent in the target quadrant during the probe test. (M) Rotarod test showing the latency to fall at day 21 after SAH. (N) Adhesive removal test showing the total time required for adhesive removal at day 21 after SAH. Data are presented as mean ± SD. ∗P < 0.05, ∗∗P < 0.01, ∗∗∗P < 0.001, and ∗∗∗∗P < 0.0001; ns, not significant.

### HDAC3 inhibition promotes oligodendrocyte development and attenuates WMI after SAH

Reclustering of the OPC/oligodendrocyte compartment identified three major lineage states: OPCs, newly formed oligodendrocytes, and mature oligodendrocytes (Fig. 4A). Subtype annotation was supported by marker expression, with Pdgfra/Cspg4 enriched in OPCs, Enpp6 in newly formed oligodendrocytes, and Mbp/Plp1/Mobp/Mag in mature oligodendrocytes (Supplementary Fig. 1A). SAH markedly reduced the proportion of mature oligodendrocytes, whereas the proportion of OPCs was increased in the RGFP966 group compared with the SAH group (Supplementary Fig. 1B). Enrichment analysis showed that central nervous system myelination and oligodendrocyte differentiation were reduced in the SAH group compared with the Sham group and partially restored by RGFP966 treatment (Fig. 4B). Consistent with these findings, myelin scores and MBP expression were increased in the RGFP966 group compared with the SAH group (Fig. 4C). Pseudotime analysis further showed that RGFP966 partially reversed SAH-induced alterations and promoted OPC development (Fig. 4D). WB demonstrated that BDNF and MBP protein levels were significantly decreased after SAH, whereas RGFP966 treatment significantly increased their expression, consistent with the IF findings (Fig. 4E–G). MRI revealed prominent brain tissue injury in the SAH group, predominantly involving the corpus callosum and striatum, whereas RGFP966 markedly alleviated these lesions (Fig. 4H and I). TEM further revealed that myelin sheaths were thinnest in the SAH + Veh group, whereas HDAC3 inhibition substantially increased myelin thickness (Fig. 4J and K). Together, these findings indicate that HDAC3 may contribute to SAH-associated WMI, whereas HDAC3 inhibition promotes oligodendrocyte development and attenuates WMI, potentially involving BDNF signaling.

**Fig. 4:**
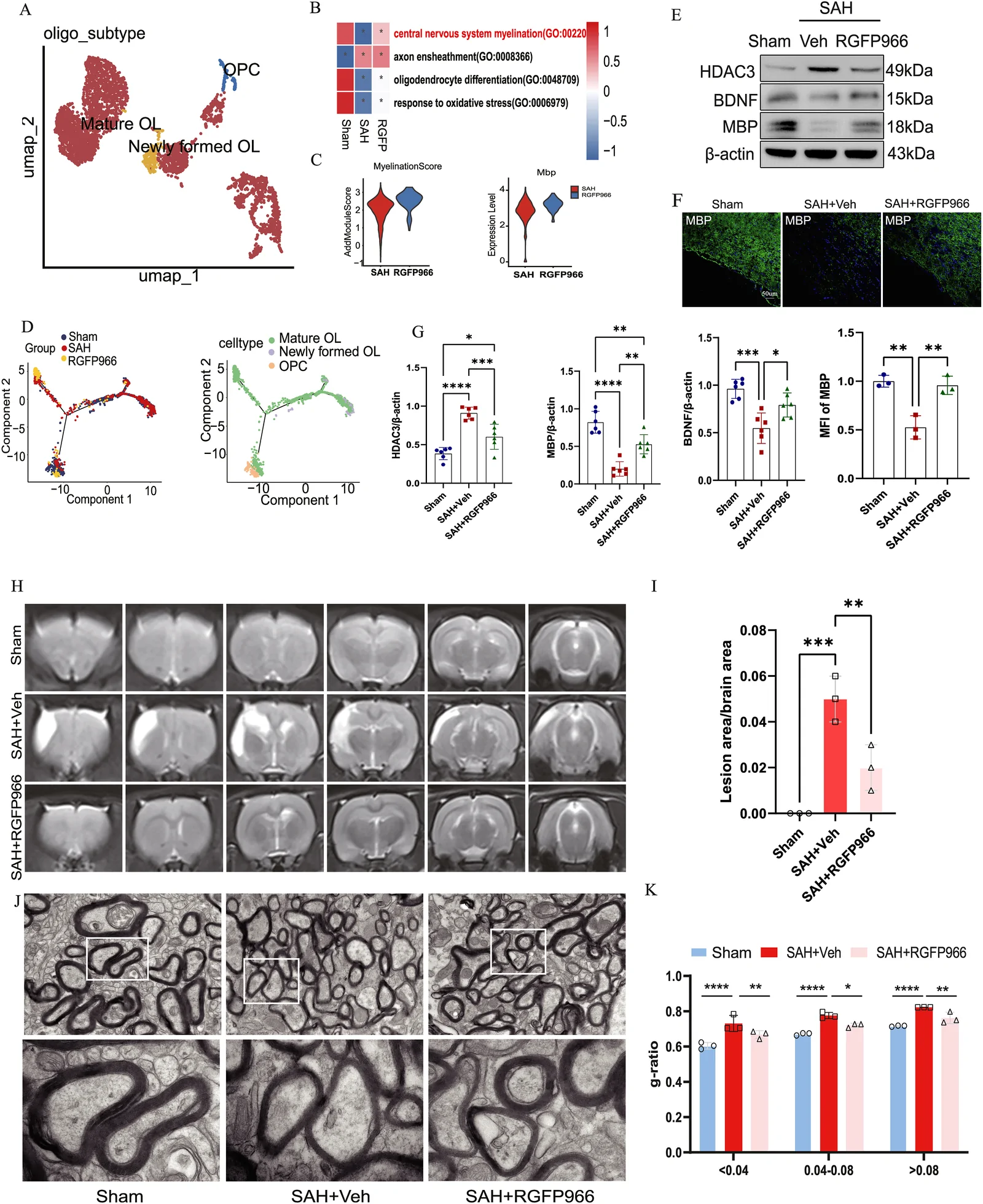
**HDAC3 inhibition promotes oligodendrocyte development and attenuates WMI after SAH** (A) UMAP visualization of the OPC/oligodendrocyte compartment showing three major lineage states: oligodendrocyte precursor cells (OPCs), newly formed oligodendrocytes (OLs), and mature OLs. (B) Enrichment analysis of biological processes associated with oligodendrocyte differentiation and myelination across the Sham, SAH, and RGFP966 groups. (C) Violin plots showing the myelination score and Mbp expression in the SAH and RGFP966 groups. (D) Pseudotime trajectory analysis of oligodendrocyte-lineage cells, colored by experimental group (left) and cell type (right). (E) Representative Western blot images of HDAC3, BDNF, and MBP expression in the Sham, SAH + Veh, and SAH + RGFP966 groups. (F) Representative immunofluorescence images of MBP in the indicated groups. (G) Quantitative analysis of HDAC3, BDNF, and MBP protein levels determined by Western blotting and MBP mean fluorescence intensity (MFI) determined by immunofluorescence. (H) Representative MR images showing brain lesions in the Sham, SAH + Veh, and SAH + RGFP966 groups. (I) Quantification of the lesion area relative to the total brain area. (J) Representative transmission electron microscopy (TEM) images of myelinated axons in the indicated groups; boxed regions are shown at higher magnification in the lower panels. (K) Quantification of the myelin g-ratio. Data are presented as mean ± SD. ∗P < 0.05, ∗∗P < 0.01, ∗∗∗P < 0.001, and ∗∗∗∗P < 0.0001.

### HDAC3 inhibition attenuates neuroinflammation and shifts microglia toward a reparative phenotype after SAH

Re-clustering identified five microglial subpopulations, including homeostatic, inflammatory, stressed, phagocytic, and ribosome biogenesis states (Fig. 5A). Subpopulation annotation was supported by the expression of canonical marker genes (Fig. 5B). SAH markedly altered microglial composition, characterized by an expansion of inflammatory microglia and a reduction in homeostatic microglia, whereas RGFP966 largely reversed these changes (Fig. 5C). Consistently, RGFP966 significantly reduced the inflammatory response score (AUCell score) following SAH (Fig. 5D). Among the microglial subpopulations, inflammatory microglia exhibited the highest inflammatory activity. Stressed microglia were also activated after SAH, whereas RGFP966 markedly reduced these scores, as assessed by AUCell and AddModuleScore (Fig. 5E). Enrichment analysis further revealed that NF-κB, TNF, Toll-like receptor, and NOD-like receptor signaling pathways were activated after SAH but suppressed by RGFP966 treatment (Fig. 5F). Pseudotime analysis indicated that SAH promoted the transition of microglia toward an inflammatory state, whereas RGFP966 inhibited this transition (Fig. 5G).

**Fig. 5:**
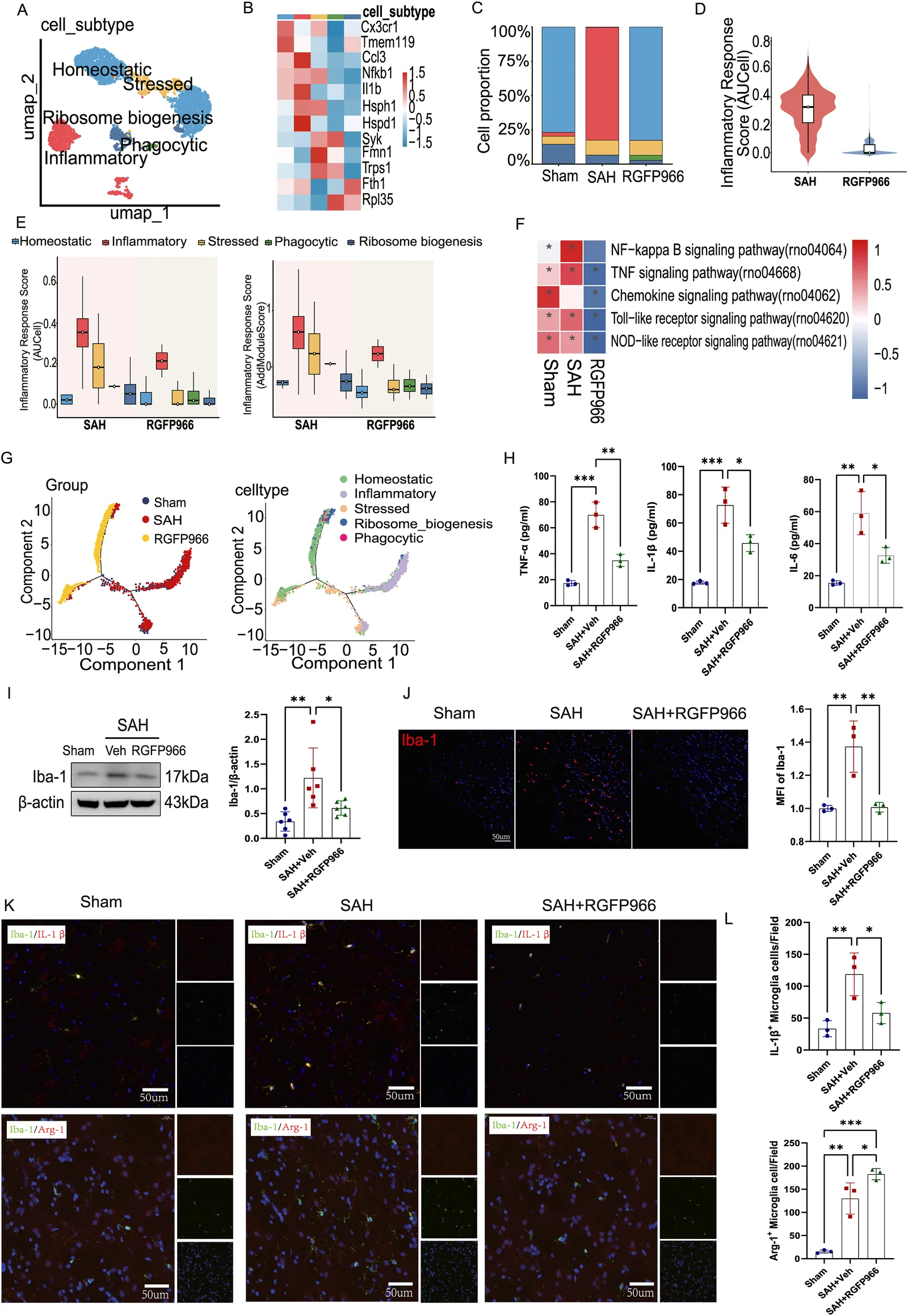
**HDAC3 inhibition attenuates neuroinflammation and shifts microglia toward a reparative phenotype after SAH** (A) UMAP visualization of five microglial subpopulations, including homeostatic, inflammatory, stressed, phagocytic, and ribosome biogenesis microglia. (B) Heatmap showing the expression of representative marker genes across microglial subpopulations. (C) Relative proportions of the five microglial subpopulations in the Sham, SAH, and RGFP966 groups. (D) Violin plots showing inflammatory response scores (AUCell scores) in the SAH and RGFP966 groups. (E) Inflammatory response scores across different microglial subpopulations in the SAH and RGFP966 groups, calculated using AUCell (left) and AddModuleScore (right). (F) Enrichment analysis of inflammation-related signaling pathways, including NF-κB, TNF, chemokine, Toll-like receptor, and NOD-like receptor signaling pathways, across the Sham, SAH, and RGFP966 groups. (G) Pseudotime trajectory analysis of microglia, colored by experimental group (left) and microglial subpopulation (right). (H) ELISA quantification of TNF-α, IL-1β, and IL-6 levels in the indicated groups. (I) Representative Western blot images and quantitative analysis of Iba-1 protein expression. (J) Representative immunofluorescence images of Iba-1 and quantitative analysis of Iba-1 mean fluorescence intensity (MFI). (K) Representative immunofluorescence images showing Iba-1/IL-1β (upper panels) and Iba-1/Arg-1 (lower panels) co-staining in the Sham, SAH, and SAH + RGFP966 groups. (L) Quantification of IL-1β-positive and Arg-1-positive microglia. Data are presented as mean ± SD. ∗P < 0.05, ∗∗P < 0.01, and ∗∗∗P < 0.001.

Consistent with the single-cell transcriptomic findings, ELISA showed that TNF-α, IL-1β, and IL-6 levels were increased after SAH and reduced by RGFP966 treatment (Fig. 5H). WB and IF further demonstrated increased IBA1 expression after SAH, which was attenuated by RGFP966 treatment (Fig. 5I and J). In addition, the numbers of both IL-1β-positive and Arg-1-positive microglia were increased after SAH. RGFP966 treatment reduced the number of IL-1β-positive microglia while further increasing the number of Arg-1-positive microglia (Fig. 5K–L). Collectively, these findings indicate that HDAC3 inhibition attenuates neuroinflammation and shifts microglia toward a reparative phenotype after SAH.

### BDNF regulates TrkB/NF-κB signaling, microglial polarization, and MBP expression after SAH

Compared with the Sham group, the SAH + Veh group showed decreased BDNF expression and TrkB phosphorylation, but increased P65 phosphorylation. Compared with the SAH + Veh group, rhBDNF treatment increased TrkB phosphorylation while decreasing P65 phosphorylation, whereas BDNF knockdown produced the opposite effects (Fig. 6A–D). Moreover, SAH increased Arg-1 and CD86 levels and decreased MBP expression compared with the Sham group. BDNF treatment further increased Arg-1, decreased CD86, and restored MBP expression, whereas BDNF knockdown decreased Arg-1 and increased CD86, accompanied by reduced MBP expression compared with the SAH + Veh group (Fig. 6 E–G).

**Fig. 6:**
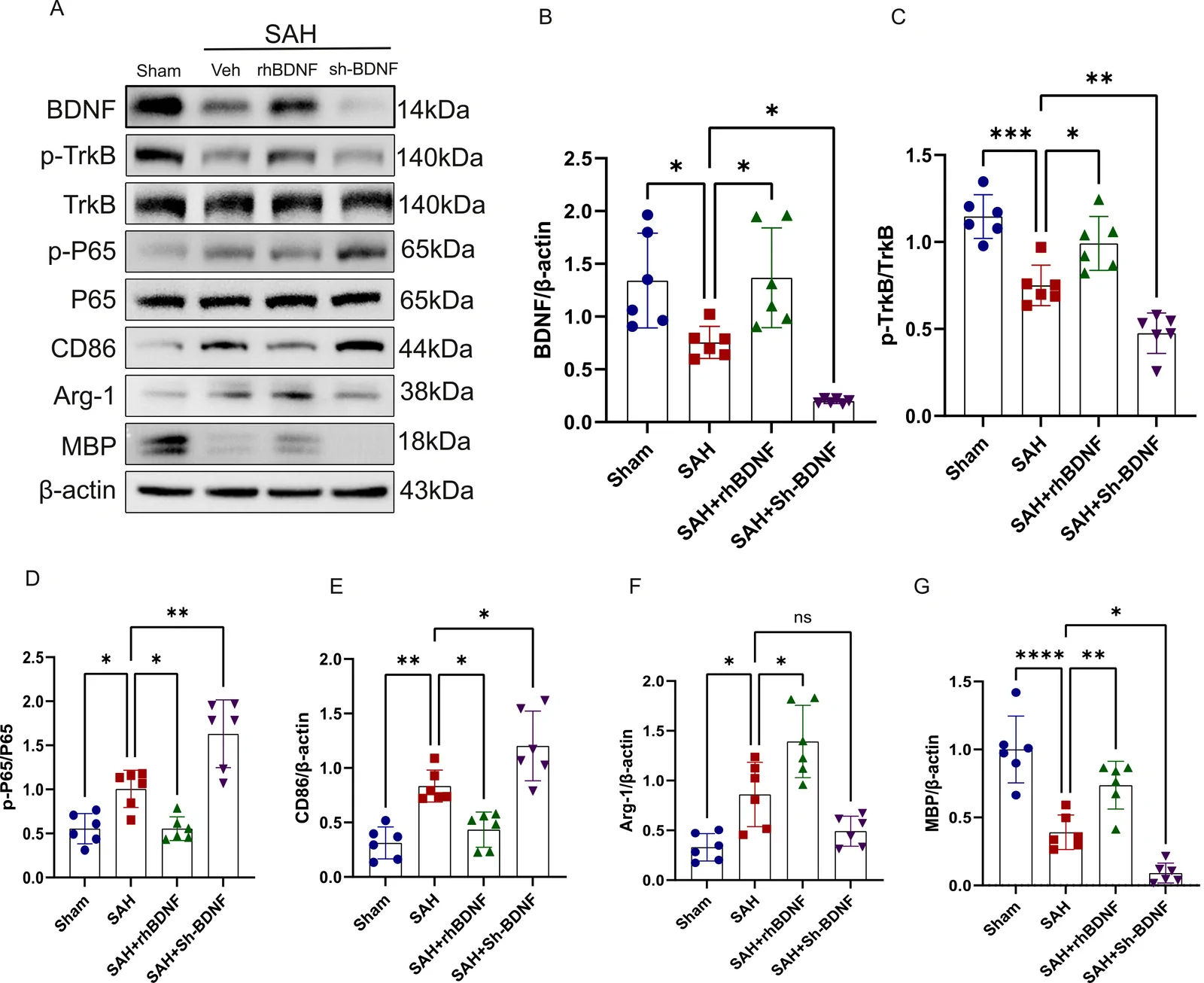
**BDNF regulates TrkB/NF-κB signaling, microglial polarization, and MBP expression after SAH** (A) Representative Western blot images of BDNF, phosphorylated TrkB (*p*-TrkB), total TrkB, phosphorylated p65 (p-p65), total p65, CD86, Arg-1, and MBP in the Sham, SAH + Veh, SAH + rhBDNF, and SAH + sh-BDNF groups. (B) Quantitative analysis of BDNF protein expression. (C) Quantification of the *p*-TrkB/TrkB ratio. (D) Quantification of the p-p65/p65 ratio. (E, F) Quantitative analysis of CD86 (E) and Arg-1 (F) protein expression. (G) Quantitative analysis of MBP protein expression. Data are presented as mean ± SD. ∗P < 0.05, ∗∗P < 0.01, ∗∗∗P < 0.001, and ∗∗∗∗P < 0.0001; ns, not significant.

### BDNF knockdown partially reversed the protective effect of HDAC3 inhibition on neurological function and WMI

Behavioral assessments, including the Morris water maze, rotarod, and adhesive removal tests, consistently demonstrated that BDNF knockdown partially reversed the beneficial effects of the HDAC3 inhibitor RGFP966 on neurological function after SAH (Fig. 7A–D). LFB staining showed that RGFP966 attenuated WMI in the corpus callosum and striatum, whereas BDNF knockdown partially reversed this effect (Fig. 7E–G). Consistently, IF analysis revealed that BDNF knockdown partially reversed the RGFP966-induced increase in MBP expression in the corpus callosum (Fig. 7H–I).

**Fig. 7:**
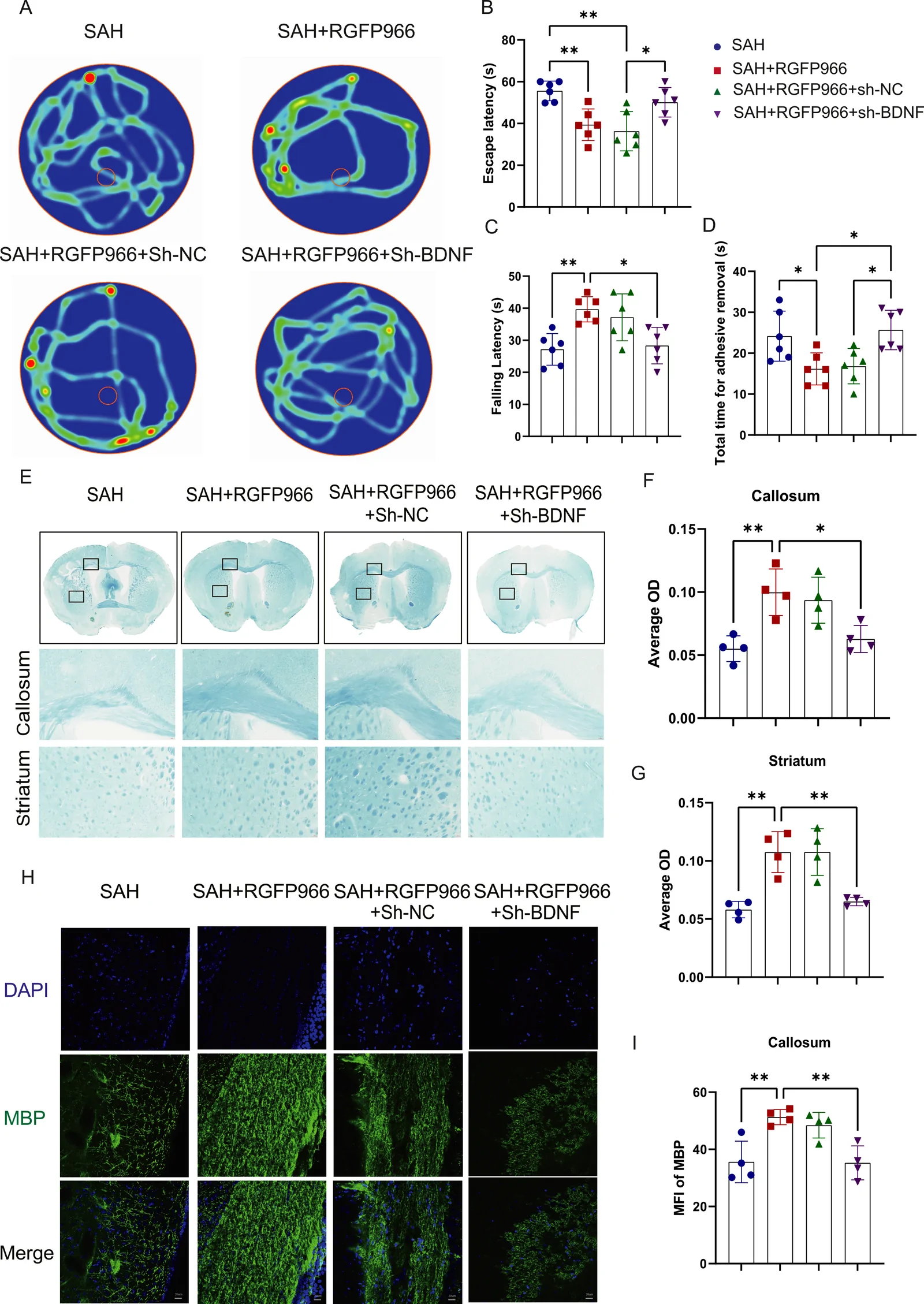
**BDNF knockdown partially reversed the protective effect of HDAC3 inhibition on neurological function and WMI** (A) Representative swimming trajectories during the hidden-platform acquisition trial of the Morris water maze in the SAH, SAH + RGFP966, SAH + RGFP966 + sh-NC, and SAH + RGFP966 + sh-BDNF groups. (B) Quantification of escape latency in the Morris water maze at 7 days after SAH. (C) Falling latency in the rotarod test at 7 days after SAH. (D) Total time for adhesive removal in the adhesive removal test at 7 days after SAH. (E) Representative images of Luxol fast blue (LFB) staining showing myelin integrity in the corpus callosum and striatum at 7 days after SAH. Boxed regions in the upper panels indicate the areas shown at higher magnification below. (F, G) Quantification of LFB staining by average optical density (OD) in the corpus callosum (F) and striatum (G). (H) Representative immunofluorescence images of myelin basic protein (MBP, green) in the corpus callosum at 7 days after SAH. (I) Quantification of the mean fluorescence intensity (MFI) of MBP in the corpus callosum. Data are presented as mean ± SD. ∗P < 0.05, ∗∗P < 0.01.

### HDAC3 inhibition alleviates neuroinflammation and WMI involving BDNF/TrkB/NF-κB signaling after SAH

KEGG enrichment analysis showed that RGFP966 markedly reversed multiple inflammation-related pathways altered after SAH. GO enrichment analysis further indicated that the effects of RGFP966 were mainly associated with inflammatory response-related biological processes, including neutrophil chemotaxis, response to lipopolysaccharide, and other stress-related processes (Supplementary Fig. 1 C–D). Cell–cell communication analysis revealed enhanced microglia–oligodendrocyte communication after SAH, which was partially reversed by RGFP966 treatment (Fig. 8 A). In microglial subpopulations, GSEA showed that SAH-induced activation of NF-κB, TNF, Toll-like receptor, and NOD-like receptor signaling pathways was suppressed by RGFP966 treatment (Fig. 8 B). In addition, compared with the SAH + RGFP966 group, the SAH + RGFP966+sh-BDNF group exhibited reduced TrkB phosphorylation, increased p65 phosphorylation, and decreased MBP expression (Fig. 8 C–D). Meanwhile, BDNF knockdown increased the proportion of pro-inflammatory (CD86-positive) microglia and decreased the proportion of reparative (Arg-1-positive) microglia (Fig. 8 E–F).

**Fig. 8:**
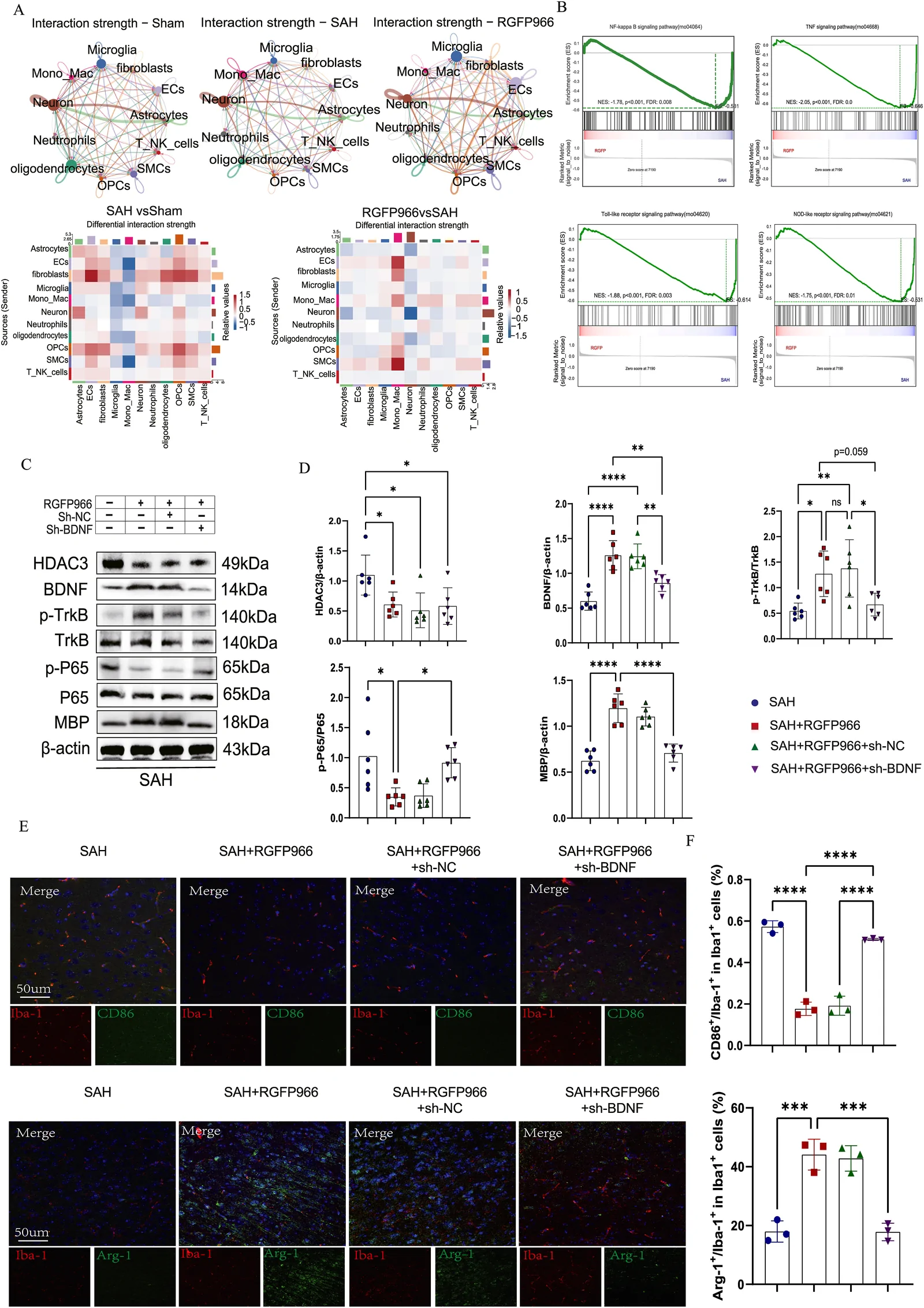
**HDAC3 inhibition alleviates neuroinflammation and WMI involving BDNF/TrkB/NF-κB signaling after SAH** (A) Cell–cell communication analysis showing the overall interaction strength among major cell types in the Sham, SAH, and RGFP966 groups (upper panels), and heatmaps showing differential interaction strength between the SAH and Sham groups and between the RGFP966 and SAH groups (lower panels). (B) GSEA of microglia showing enrichment of inflammation-related signaling pathways, including NF-κB, TNF, Toll-like receptor, and NOD-like receptor signaling pathways. (C) Representative Western blot images of HDAC3, BDNF, phosphorylated TrkB (*p*-TrkB), total TrkB, phosphorylated p65 (p-p65), total p65, and MBP in the SAH, SAH + RGFP966, SAH + RGFP966+sh-NC, and SAH + RGFP966+sh-BDNF groups. (D) Quantitative analysis of HDAC3, BDNF, *p*-TrkB/TrkB, p-p65/p65, and MBP protein levels. (E) Representative immunofluorescence images showing Iba-1/CD86 (upper panels) and Iba-1/Arg-1 (lower panels) co-staining in the indicated groups. Scale bar = 50 μm. (F) Quantification of the proportions of CD86-positive and Arg-1-positive cells among Iba-1-positive microglia. Data are presented as mean ± SD. ∗P < 0.05, ∗∗P < 0.01, ∗∗∗P < 0.001, and ∗∗∗∗P < 0.0001; ns, not significant.

## Discussion

In the present study, we identified HDAC3 as a dynamically regulated injury-associated molecule after subarachnoid hemorrhage and demonstrated that pharmacological inhibition of HDAC3 confers sustained neuroprotective effects (Fig. 9). Specifically, HDAC3 was markedly increased after SAH in both patient cerebrospinal fluid and rat brain tissue, whereas MBP showed an opposite temporal pattern. HDAC3 inhibition with RGFP966 improved short and long-term neurological performance, attenuated structural white matter damage, reshaped microglial inflammatory states, and altered oligodendrocyte-lineage responses after injury. Mechanistically, these protective effects were associated with restoration of BDNF/TrkB signaling and suppression of NF-κB-related inflammatory activation. Collectively, these findings support HDAC3 as an important regulator of the neuroinflammation–WMI axis after SAH.

**Fig. 9:**
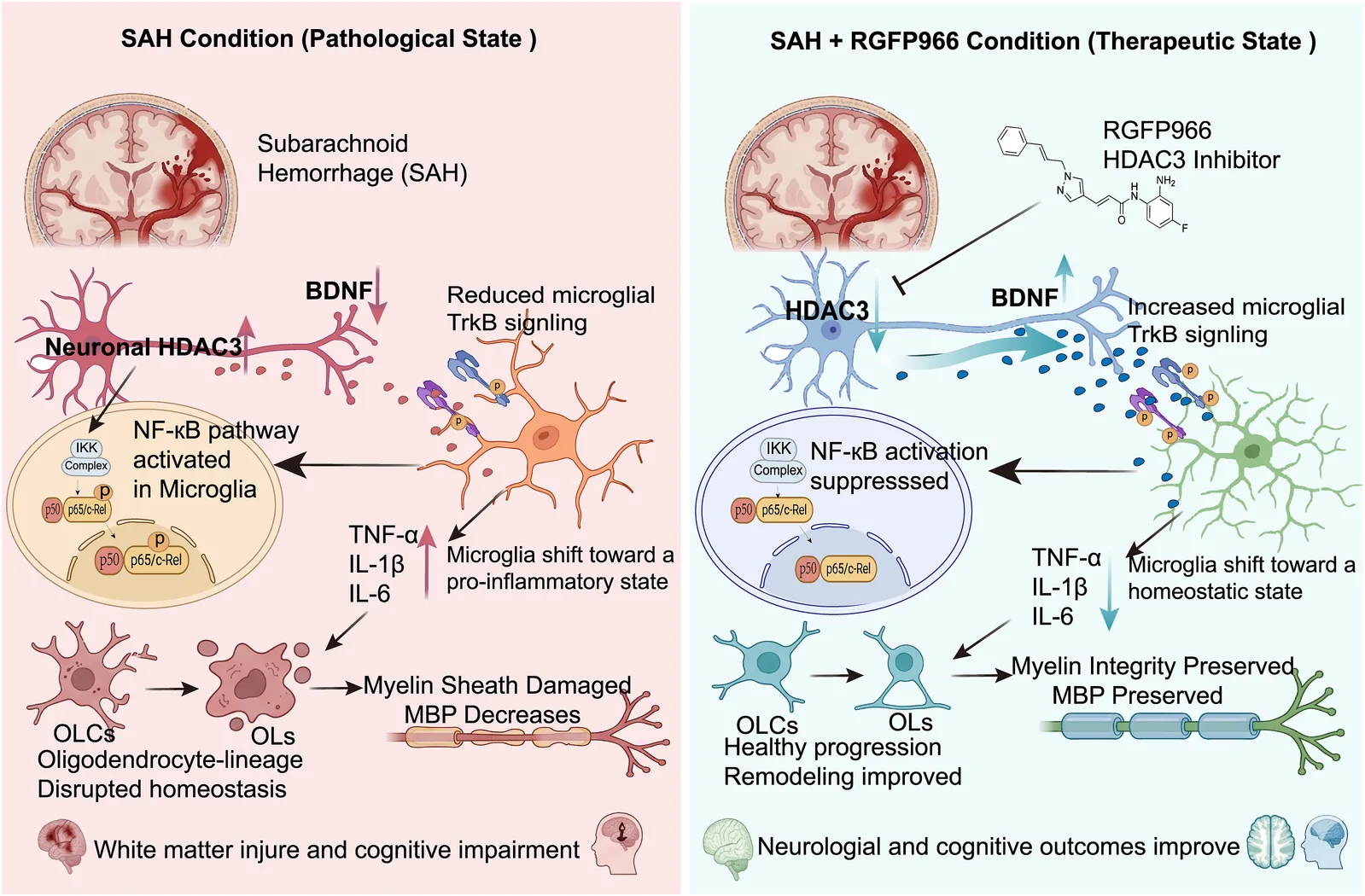
**Schematic illustration of HDAC3 inhibition attenuating neuroinflammation and white matter injury after SAH** HDAC3 inhibition promotes microglial homeostasis and preserves myelin integrity by modulating BDNF/TrkB/NF-κB signaling, thereby improving neurological and cognitive outcomes after SAH.

HDAC3 is a key epigenetic regulator involved in transcriptional repression, inflammatory signaling, and responses to neural injury, and has been recognized as a potential therapeutic target in neurodegenerative diseases [34], [35], multiple sclerosis [36], and ischemic stroke [37]. However, its role in hemorrhagic stroke has been scarcely investigated. Our study extends this concept to the context of SAH and suggests that HDAC3 may participate in both early tissue injury and subsequent white matter remodeling after SAH. Recent studies have shown that HDAC3 is closely associated with neuroinflammation, axonal injury, and white matter pathology. Particularly relevant to our findings, pharmacological inhibition of HDAC3 has been reported to attenuate microglial activation and pro-inflammatory cytokine release, thereby alleviating oligodendrocyte developmental impairment and defective myelination [38]. In addition, previous studies have demonstrated that microglia-specific deletion of HDAC3 suppresses pro-inflammatory microglial responses and neuroinflammation, mitigates TBI-induced axonal and myelin injury, and ultimately improves long-term neurological recovery in mice [13]. Another study showed that HDAC3-deficient microglia promote post-stroke remyelination by recruiting macrophages to accelerate myelin debris clearance [14]. Most previous studies have focused primarily on the role of microglial HDAC3. In contrast, our single-cell transcriptomic analysis revealed that HDAC3 was predominantly expressed in neurons (Supplementary Fig. 1E–F), suggesting that neuron-specific regulation of HDAC3 may be of particular protective significance. One potential mechanism is that neuronal HDAC3 may modulate neuron–microglia communication, thereby contributing to the regulation of neuroinflammation.

One important finding of the present study is that HDAC3 increased dynamically after SAH and showed an inverse temporal relationship with MBP expression. More importantly, pharmacological inhibition of HDAC3 not only restored MBP expression and alleviated SAH-induced axonal and myelin injury histologically, but also improved long-term cognitive impairment in SAH rat These findings suggest that the protective effects of RGFP966 are not confined to acute anti-inflammatory actions, but may also extend to preservation of white matter integrity and promotion of long-term functional recovery. This differs from earlier studies that mainly focused on early brain injury [39], and further underscores the therapeutic potential of targeting HDAC3.

Mechanistically, the regulatory effect of HDAC3 inhibition on microglial responses appears to be particularly important, which may explain why previous studies have mainly focused on microglial HDAC3. Microglia are among the earliest activated cells after SAH and play a central role in shaping the inflammatory microenvironment that drives SBI during the acute phase of SAH [40]. Excessive or sustained microglial activation can aggravate tissue damage through the release of pro-inflammatory cytokines, reactive oxygen species, and other injury-related mediators [41] In the present study, single-cell transcriptomic analysis revealed marked remodeling of the microglial landscape after SAH, characterized by a reduction in homeostatic microglia and an expansion of inflammatory microglia. RGFP966 largely reversed these changes, increasing the proportion of homeostatic microglia while reducing inflammatory microglia. Consistently, inflammatory response scores were decreased, and inflammation-related pathways, including NF-κB, TNF, Toll-like receptor, and NOD-like receptor signaling, were suppressed. These transcriptomic findings were further supported by conventional experimental data showing reduced Iba-1 expression and lower levels of TNF-α, IL-1β, and IL-6. Taken together, these results indicate that HDAC3 inhibition attenuates maladaptive microglial inflammatory activation after SAH.

In addition to microglia, HDAC3 inhibition also reshaped oligodendrocyte-lineage states and myelination-related transcriptional programs. Re-clustering analysis of the oligodendrocyte lineage identified distinct states, including OPCs, newly formed oligodendrocytes, and mature oligodendrocytes. SAH disrupted the relative distribution of these cellular states, indicating impaired oligodendrocyte-lineage homeostasis after injury. RGFP966 further altered lineage composition and regulated programs related to myelination, oligodendrocyte differentiation, and axonal ensheathment, which is consistent with previous findings [42]. At the same time, myelin-related genes such as Mbp, Plp1, and Mobp also showed a trend toward preservation or recovery following HDAC3 inhibition. These findings suggest that HDAC3 inhibition may promote adaptive remodeling of the oligodendrocyte lineage after SAH. Nevertheless, these oligodendrocyte-lineage changes should be interpreted cautiously. The observed transcriptional alterations do not necessarily indicate that oligodendrocyte maturation has been fully restored to the pre-injury baseline. A more plausible explanation is that HDAC3 inhibition promotes reparative remodeling under pathological conditions. Therefore, the white matter-protective effects of HDAC3 inhibition may derive not only from direct actions on oligodendrocyte-lineage cells, but also from improvements in the surrounding inflammatory microenvironment. Given that oligodendrocytes and OPCs are highly sensitive to inflammatory mediators, suppression of microglia-driven neuroinflammation may create a more favorable environment for myelin maintenance or repair. Moreover, a recent study reported that HDAC3 inhibition alleviates oligodendrocyte-lineage dysfunction and preserves myelin integrity, possibly by reducing ferroptosis in white matter cells through regulation of iron metabolism and lipid peroxidation [19].

Another major finding of this study is that the BDNF/TrkB/NF-κB axis is involved in HDAC3-mediated neuroprotection. BDNF is a critical neurotrophic factor that plays key roles in neuronal survival, synaptic plasticity, and glial regulation, and has also been implicated in myelin maintenance and repair after CNS injury. Previous studies have shown that BDNF is a transcriptional target suppressed by HDAC3, and that HDAC3 inhibition can exert neuroprotective effects by upregulating neuroprotective genes such as BDNF and Npas4 [43]. However, the mechanism by which BDNF modulates microglial inflammatory signaling remains unclear. In the present study, both BDNF expression and TrkB phosphorylation were decreased after SAH, whereas HDAC3 inhibition restored BDNF levels. Moreover, exogenous BDNF supplementation increased TrkB phosphorylation, reduced p-P65 levels, promoted a shift of microglia toward a more anti-inflammatory phenotype, and increased MBP expression, whereas BDNF knockdown produced the opposite effects. These findings suggest that BDNF/TrkB signaling lies upstream of microglial NF-κB-related inflammatory activation and contributes to the anti-inflammatory and white matter-protective effects mediated by HDAC3 inhibition. It should also be noted that BDNF/TrkB may represent only one of the downstream pathways through which HDAC3 regulates neuroinflammation. HDAC3 inhibition may also exert its effects through other BDNF-independent mechanisms. Previous studies have shown that multiple signaling pathways, including Stat1/Stat3 [44], [45], Nrf1/Nrf2 [46], and cGAS/STING [47], can also be influenced by HDAC3 and converge on NF-κB inflammatory signaling.

From a translational perspective, our findings further suggest that HDAC3 may have both biomarker value and therapeutic relevance. Elevated HDAC3 and reduced MBP levels in the cerebrospinal fluid of patients with SAH indicate that HDAC3-associated signaling may reflect an ongoing injury process during the clinical course of SAH. RGFP966 is a highly selective HDAC3 inhibitor that can cross the blood–brain barrier, and its safety, dosing, and neuroprotective effects have been validated in experimental stroke models [48], [49]. Our study further highlights the neuroprotective effects and potential translational value of RGFP966 in a rat model of SAH. Given that effective interventions for SBI and long-term functional impairment after SAH remain limited, targeting HDAC3-dependent inflammatory and WMI pathways may represent a promising therapeutic strategy worthy of further investigation.

Several limitations of this study should be acknowledged. First, although our findings support the involvement of BDNF/TrkB/NF-κB signaling, the precise epigenetic mechanism by which HDAC3 regulates BDNF transcription was not directly investigated. Moreover, while BDNF dependency was examined using a knockdown strategy, TrkB dependency was not directly established through pharmacological or genetic manipulation. Further studies are therefore required to define the causal contribution of TrkB to this signaling axis. Second, only one sample per group was included in the single-cell RNA sequencing analysis, which may have increased the potential influence of inter-individual variability. Third, our assessment of microglial polarization relied in part on traditional markers, such as IL-1β and Arg-1, which may not fully capture the complexity and heterogeneity of microglial states in vivo. Fourth, direct target engagement of RGFP966 was not assessed in the present SAH model, and dose-response analyses were not performed. Future studies incorporating measurements of HDAC3 activity and histone acetylation, together with dose-response analyses, are needed to further define the pharmacodynamic effects and optimal dosing regimen of RGFP966 after SAH. Finally, the 28-day observation period may be insufficient to fully characterize long-term white matter remodeling and neurological recovery, and longer-term studies are warranted to determine the durability of the observed protective effects.

## Conclusion

In summary, our study identifies HDAC3 as an important but previously underappreciated regulator of neuroinflammation and WMI after SAH. Pharmacological inhibition of HDAC3 attenuated microglial inflammatory activation, reshaped oligodendrocyte-lineage responses, preserved myelin integrity, and improved short and long-term neurological outcomes. These protective effects were associated with restoration of BDNF/TrkB signaling and suppression of NF-κB activation. Collectively, our findings suggest that HDAC3 may represent a promising therapeutic target for limiting SBI and promoting white matter repair after SAH.

## Author contributions

Conceptualization, Yi Huang and Xiang Gao; Data curation, Hongqiao Yang, Ziliang Hu, Hao Zhuang, Siqi Chen, Shengjun Zhou, Meng Sun, Lifei Lian, Chenhui Zhou; methodology, Hongqiao Yang, Ziliang Hu, Hao Zhuang, Siqi Chen, Shengjun Zhou, Meng Sun; writing—original draft preparation, Hongqiao Yang, Ziliang Hu, Hao Zhuang; writing—review and editing, Xiang Gao and Yi Huang; project administration, Yi Huang; Funding acquisition, Shengjun Zhou, Xiang Gao and Yi Huang. All authors have read and agreed to the published version of the manuscript.

## Ethics approval and consent to participate

Clinical trial number: not applicable. Human subject investigations received by the Ethics Review Committee of the First Affiliated Hospital of Ningbo University (Approval No: 2024113 A). Animal experimentation was authorized by Ningbo University's Animal Ethics Committee (Approval No: NBU20230394).

## Availability of data and materials

The Sc-RNA seq data reported in this paper have been deposited in the National Genomics Data Center, China National Center for Bioinformation (accession no. OMIX020008). Related data and analytical pipelines will be available from the corresponding authors on reasonable request.

## Consent for publication

Not applicable.

## Declaration of generative Al and Al-assisted technologies in the writing process

During the preparation of this work, the authors used Gemini in order to improve the linguistic quality and grammatical accuracy of the manuscript. After using this tool, the authors strictly reviewed and edited the content as needed and take full responsibility for the scientific integrity and accuracy of the final publication.

## Funding

This study was supported by grants from 10.13039/501100004731Zhejiang Provincial Natural Science Foundation (LMS26H090007), Ningbo Top Medical and 10.13039/100005622Health Research Program (2022020304). 10.13039/100007834Ningbo Natural Science Foundation (2023J019), Ningbo Youth Science and Technology Innovation Leading Talent Project (2025QL015).

## Competing interests

The authors declare no conflict of interest.
