# Supplementary material for: HDAC3 inhibition alleviates neuroinflammation and white matter injury after subarachnoid hemorrhage through modulation of BDNF/TrkB/NF-κB signaling
Source: Neurotherapeutics. 2026 Oct 28;24(1):e01099. doi: 10.1016/j.neurot.2026.e01099 (PMC13738182; doi:10.1016/j.neurot.2026.e01099)
Supplement: Supplementary file 1 [file mmc1.docx]

**Supplementary Figure 1. Single-cell transcriptomic analyses of oligodendrocyte lineage states and RGFP966-associated transcriptional changes after SAH.**
(A) Dot plot of representative marker genes supporting the annotation of oligodendrocyte lineage states, including OPCs, newly formed oligodendrocytes, and mature oligodendrocytes. (B) Stacked bar plot showing the proportional distribution of oligodendrocyte lineage states across the Sham, SAH, and RGFP966 groups. (C) KEGG enrichment analysis of downregulated genes in the RGFP966 versus SAH comparison. (D) GO enrichment analysis of downregulated genes in the RGFP966 versus SAH comparison. (E) Dot plot showing the expression patterns of Hdac3 and Mbp across major cell types in the Sham, SAH, and RGFP966 groups. (F) UMAP feature plots showing the distribution of Hdac3 and Mbp expression in the Sham, SAH, and RGFP966 groups.
